# Hyperuricemia and untreated gout are poor prognostic markers among those with a recent acute myocardial infarction

**DOI:** 10.1186/ar3684

**Published:** 2012-01-17

**Authors:** Eswar Krishnan, Bhavik J Pandya, Bharathi Lingala, Ali Hariri, Omar Dabbous

**Affiliations:** 1 Department of Medicine, Stanford University School of Medicine, 1000 Welch Rd, Suite 203, Palo Alto, CA 94304, USA; 2 Takeda Pharmaceuticals International, Inc. One Takeda Parkway, Deerfield, IL 60015, USA; 3 Takeda Pharmaceuticals North America, Inc. One Takeda Parkway, Deerfield, IL 60015, USA

## Abstract

**Introduction:**

Patients with a history of myocardial infarction (MI) are often at risk for complications, including subsequent MI and death. Use of prognostic markers may aid in preventing these poor outcomes. Hyperuricemia is associated with increased risk for coronary heart disease (CHD) and/or mortality; however, it is unknown if serum urate (sUA) levels predict outcomes in patients with previous MI. The purpose of this study was to assess hyperuricemia as a biomarker of CHD outcomes in such patients.

**Methods:**

These were post hoc analyses of datasets from the Aspirin Myocardial Infarction Study, a 1:1 randomized, double-blind clinical trial, conducted from 1975 to 1979, that examined mortality rates following daily aspirin administration over three years in individuals with documented MI. The primary outcome measures were all-cause death, CHD mortality, coronary incidence, and stroke by quartile of baseline sUA. A sub-analysis of all outcome measures in the presence or absence of gouty arthritis was also performed.

**Results:**

Of 4,524 enrolled participants, data on 4,352 were analyzed here. All outcomes were greatest for patients in the fourth sUA quartile. In multivariate regression models, the hazard ratios (HR) for patients in the highest quartile were 1.88 for all-cause mortality (95% confidence interval (CI), 1.45 to 2.46), 1.99 for CHD mortality (95% CI, 1.49 to 2.66), and 1.36 for coronary incidence (95% CI, 1.08 to 1.70). Participants with untreated gout had an adjusted hazard ratio ranging from 1.5 to 2.0 (all *P *< 0.01) for these outcomes. Participants with gout who were receiving treatment did not exhibit this additional risk.

**Conclusions:**

sUA and untreated gout may be independent prognostic markers for poor all-cause and CHD mortality in patients with recent acute MI.

## Introduction

In 2010, the estimated annual incidence of myocardial infarction (MI) in the US was 610,000 new attacks and 325,000 recurrent attacks [1]. Following MI, 16% and 22% of men and women, respectively, between the ages of 40 and 69 years, experience a recurrent MI or fatal coronary heart disease (CHD) [1]. Since the underlying pathophysiology of disease in patients with acute coronary syndrome (ACS) varies widely, accurate risk stratification to determine appropriate management and improve outcomes is essential. As such, the use of prognostic biomarkers may facilitate the ability to anticipate complications following MI and provide timely preventive care to at-risk individuals [2]. For example, increased concentration of cardiac troponin can predict adverse events following MI or ACS [3]. Other markers of post-MI or post-ACS outcomes include N-terminal pro-B-type natriuretic peptide, inflammatory markers such as C-reactive protein (CRP), monocyte chemoattractant protein-1, and interleukin-6 [2,4,5].

Hyperuricemia is known to be correlated with a greater risk for coronary artery calcification, a surrogate measure of coronary atherosclerosis [6]. Both asymptomatic hyperuricemia and gout have been identified as independent risk factors for the development and progression of cardiovascular disease (CVD), CVD mortality, and all-cause mortality, in a variety of populations [7,8]. However, it is unknown whether this excess risk extends beyond the initial coronary event. In this study, we hypothesized that mortality rates increase with serum urate levels (sUA) in patients with established CHD. To test this, we analyzed data from a three-year prospective, observational study that monitored levels of sUA in patients with histories of MI.

## Materials and methods

### Study design

This study was performed as a post hoc analysis using datasets from the Aspirin Myocardial Infarction Study (AMIS) (ClinicalTrials.gov Identifier: NCT00000491), a National Heart, Lung and Blood Institute (NHLBI)-sponsored 1:1 randomized, double-blind clinical trial. It examined the hypothesis that daily administration of 1 g of aspirin orally in 2 divided doses to individuals with documented CHD would result in significantly reduced mortality over a three-year period [9]. Ethical approval was obtained at each of the enrolling sites of this study. All participants provided informed consent.

The design of the AMIS has been described previously [9]. In brief, 4,524 persons between 30 and 69 years of age were randomized 8 weeks to 5 years after a qualifying MI to receive either 1 g aspirin per day (*n *= 2,267) or placebo (*n *= 2,257) over a 13-month period at 30 clinical centers starting in 1974. Main inclusion criterion was presence of documented MI. The main exclusion criteria included history of aspirin intolerance, severe peptic ulcer disease, previous cardiovascular (CV) surgery, uncontrolled hypertension, and use of anticoagulants, aspirin, dipyridamole, or sulfinpyrazone. The study also followed participants for a minimum of three years for mortality outcomes and non-fatal CHD events. Subcommittees of investigators adjudicated CV and mortality outcome assessments in a standardized manner. Enrollees reported to clinical centers every four months except the first post-randomization visit that occurred at the end of month one. Participants were educated about avoiding aspirin and aspirin-containing over-the-counter medications; acetaminophen was suggested for analgesic use. The last study visit in the trial was August 6, 1979, when all study medications were discontinued and treatment assignment was revealed.

### Outcome measures

The present analysis included data from all participants with information on sUA at baseline (*n *= 2,169 in the aspirin group; *n *= 2,183 in the placebo group; *n *= 4,352 overall). The analysis also utilized annual follow-up data from all participants. The outcome definitions were similar to those of AMIS: 1) all-cause mortality; 2) CHD mortality, defined as death from definite MI documented by electrocardiogram and enzyme changes or autopsy, deaths suspected to be due to MI, and sudden unexpected deaths (death within one hour of onset of symptoms, excluding deaths from accidents, homicides, or suicides); 3) coronary incidence, defined as CHD mortality or non-fatal MI; and 4) fatal or non-fatal stroke [9].

Gouty arthritis, defined as a lifetime history of self-reported physician diagnosis of gout at baseline or any time during the study was another covariate of interest. This case definition is often used in epidemiological studies and has been found to have excellent sensitivity [10-12]. The specificity may not be high. Nevertheless, as long as this type of measurement error is non-differential (that is, the magnitude and direction of error is the same in all comparison groups) its impact on study analyses will be negligible. Because participants seldom had gout flares and/or confirmation by joint aspiration by the study physician at the time of study visits, case definition of gouty arthritis included use of gout medications. Gout medications included allopurinol, probenecid, and colchicine (dosage information not available); sulfinpyrazone use was not permitted in the trial. Because of these data, outcomes by gout status were investigated as a secondary analysis in which the participant group without gout was compared to a) participants with gout who received treatment, and b) participants with untreated gout.

### Analytical approach

The hypothesis tested for this study was that hyperuricemia and gout are risk factors for CHD and all-cause mortality among those with existing CAD. All data for the two study arms were analyzed separately prior to pooling, as regular aspirin use may have statistical and biological impacts on sUA concentration, gout and the study outcomes. Because the distribution of sUA is systemically different between men and women, quartile cutoffs were defined separately for each, based on baseline measurements. For the aspirin group, quartile 1 was defined as 2.5 to 4.5 mg/dL for men, 1.4 to 5.25 mg/dL for women; quartile 2 was 4.53 to 5.37 mg/dL for men, 5.27 to 6.03 mg/dL for women; quartile 3 was 5.38 to 6.45 mg/dL for men, 6.05 to 7.0 mg/dL for women; and quartile 4 was 6.47 to 10.45 mg/dL for men, 7.02 to 12.52 mg/dL for women. For the placebo group, quartile 1 was defined as 2.38 to 4.48 mg/dL for men, 1.83 to 5.28 mg/dL for women; quartile 2 was 4.5 to 5.42 mg/dL for men, 5.3 to 6.03 mg/dL for women; quartile 3 was 5.47 to 6.48 mg/dL for men, 6.05 to 6.97 mg/dL for women; and quartile 4 was 6.52 to11.05 mg/dL for men, 6.98 to 12.12 mg/dL for women. The corresponding figures in SI units (μmol/L) can be calculated by multiplying by 59.48. The covariates used in the present analyses were age, gender, African American ethnicity, body mass index, blood pressure/hypertension, smoking, diabetes status, physical activity measure, hyperlipidemia, hypertriglyceridemia, renal function, and other non-steroidal anti-inflammatory drug use. Participants who were actively treated for diabetes, hypertension, and hyperlipidemia were considered to have those conditions.

Chi-square tests were used to assess statistical significance between cumulative incidences of outcomes by study group. For primary analyses, we used multivariable Cox regression models to generate hazard ratios (HR) after ensuring the condition of proportionality was met. In these regressions, time to the event was modeled as a function of baseline sUA concentration. Since the lifetime duration of hyperuricemia was unknown, we recognized that the Cox models may be subject to left censoring and the data were reanalyzed using logistic regression models. The results of logistic regression models did not differ from Cox models--the latter are presented here.

## Results

### Study participants

Baseline characteristics of the study group stratified by quartile of sUA concentration are presented in Table 1. The population receiving aspirin had a mean age of 54.9 ± 8.1 years at randomization, and consisted of 88.5% men and 6.4% African Americans. Individuals receiving placebo had a mean age of 54.9 ± 7.9 years, and consisted of 89.6% men and 5.9% African Americans. The mean age at first and most recent MI was 52.7 years and 53.2 years, respectively.

**Table 1:** Baseline characteristics of participants in the AMIS Study (Number = 4,352)

	Aspirin group					Placebo group				
	
	Quartile 1	Quartile 2	Quartile 3	Quartile 4	Total	Quartile 1	Quartile 2	Quartile 3	Quartile 4	Total
**sUA range, mg/dL men**	2.5-4.5	4.52-5.37	5.38-6.45	6.47-10.45	2.50-10.45	2.38-4.48	4.5-5.42	5.47-6.48	6.52-11.05	2.38-11.05
**women**	1.4-5.25	5.27-6.03	6.05-7	7.02-12.52	1.40-12.52	1.83-5.28	5.3-6.03	6.05-6.97	6.98-12.12	1.83-12.12
**Number**	543	541	541	544	2,169	547	539	551	546	2,183
**Mean sUA, mg/dL (SD)**	4.55 (0.57)	5.55 (0.34)	6.44 (0.34)	8.05 (0.99)	6.15 (1.43)	4.59 (0.57)	5.61 (0.3)	6.43 (0.32)	7.93 (0.91)	6.14 (1.35)
**Demographics**										
Age at first MI, years (SD)	52.6 (8.0)	52.5 (8.0)	52.6 (8.3)	52.8 (8.2)	52.6 (8.1)	53.0 (7.6)	52.7 (8.1)	52.5 (8.0)	52.7 (7.9)	52.7 (7.9)
Age at most recent MI, years (SD)	53.1 (7.8)	53.1 (7.9)	53.1 (8.3)	53.5 (8.0)	53.2 (8.0)	53.5 (7.6)	53.1 (8.0)	52.9 (7.9)	53.4 (7.8)	53.2 (7.8)
Age at randomization, years (SD)	54.8 (8.0)	54.8 (8.0)	54.8 (8.4)	55.1 (8.1)	54.9 (8.1)	55.2 (7.6)	54.8 (8.1)	54.5 (8.0)	55.0 (7.9)	54.9 (7.88)
Body mass index, kg/m^2 ^(SD)	24.8 (3.2)	25.6 (3.3)	26.1 (3.2)	27.1 (3.7)	25.9 (3.5)	25.1 (3.1)	25.6 (3.2)	26.4 (3.4)	26.8 (3.8)	26.0 (3.5)
Men, %	88.8	88.2	88.4	88.6	88.5	89.8	89.4	89.7	89.7	89.6
African American, %	5.7	4.3	6.7	9	6.4	7.1	5.9	3.1	7.5	5.9
**Physical examination data**										
Sedentary lifestyle, %	16.7	16.3	17.4	21.3	17.9	16.8	15.6	15.4	20.9	17.2
Alcohol use^a^, %	88.4	84.7	82.1	82	84.3	88.1	84.6	85.3	80.6	84.7
Smoker^b^, %	40.7	40.1	31.8	29.6	35.5	39.9	37.3	31.2	32.8	35.3
Heavy smoker^c^, %	10.1	10.3	12.1	10	10.6	7.2	5.8	15.7	11.4	9.8
Systolic blood pressure, mmHg (SD)	126.4 (15.6)	126.1 (15.4)	129.0 (16.6)	129.6 (16.5)	127.8 (16.1)	127.5 (17.1)	127.1 (15.1)	128.5 (15.5)	129.5 (16.9)	128.2 (16.2)
Diastolic blood pressure, mmHg (SD)	78.3 (9.4)	78.9 (8.9)	79.6 (9.2)	81.7 (9.8)	79.6 (9.4)	79.2 (9.3)	78.9 (8.9)	80.3 (9.4)	81.9 (10.0)	80.1 (9.5)
**Laboratory data**										
Total glucose, mg/100 ml (SD)	148.8 (56.8)	155.4 (57.7)	158.4 (50.1)	171.2 (49.9)	158.6 (54.3)	159.1 (67.5)	156.7 (53.3)	160.1 (50.5)	168.9 (52.2)	161.2 (56.3)
Total cholesterol, mg/dL (SD)	230.4 (44.7)	235.7 (43.3)	240.4 (43.2)	243.2 (49.0)	237.7 (45.5)	234.0 (43.2)	235.1 (42.4)	240.2 (41.8)	241.2 (42.5)	237.7 (42.5)
Triglycerides, mg/dL (SD)	160.7 (130.0)	173.3 (101.6)	190.6 (149.1)	228.7 (192.2)	189.9 (159.2)	153.3 (86.2)	162.7 (97.0)	191.5 (116.6)	230.1 (169.3)	185.2 (125.7)
**Comorbidities, %**										
Number of prior MI										
1	88.4	86.7	86.1	84.2	86.4	86.1	88.7	90.0	83.0	86.9
2	10.1	12.4	12.6	13.6	12.2	11.3	10.4	8.5	14.7	11.2
≥ 3	1.5	0.9	1.3	2.2	1.5	2.6	0.9	1.5	2.4	1.8
History of diabetes	13.4	8.1	9.2	8.8	9.9	17	10.4	7.8	10.1	11.3
History of renal impairment	4.2	4.6	5.2	6.6	5.2	3.7	4.3	4.9	6.8	4.9
Gout flares^d^	3.1	4.4	4.3	8.3	5.0	3.5	2.2	4.5	7.3	4.4
**Medications, %**										
Gout medications^d, e^	4.2	4.1	3.1	5.0	4.1	3.3	2.0	4.5	4.8	3.7
Non-aspirin NSAIDs	1.5	1.7	0.4	1.8	1.3	0.7	0.9	0.9	1.3	1.0
Anti-diabetic medications	7.9	2.8	3.5	3.3	4.4	8.0	4.8	3.4	5.7	5.5
Blood pressure medications	5.7	8.3	8.9	18.4	10.3	6.4	7.8	8.5	16.7	9.8
Lipid-lowering medications	5.3	5.7	4.6	4.6	5.1	4.9	3.9	5.4	3.8	4.5
**Follow-up time, years (SD)^f^**	2.9 (0.5)	2.9 (0.5)	2.8 (0.5)	2.8 (0.5)	2.8 (0.5)	2.8 (0.5)	2.8 (0.5)	2.9 (0.5)	2.8 (0.6)	2.8 (0.5)
Minimum (years)	1.0	0.9	0.9	0.9	0.9	0.90	0.90	0.8	0.9	0.8
Maximum (years)	3.2	3.2	3.2	3.2	3.2	3.2	3.2	3.2	3.1	3.2

^a ^< 1.5 oz/day; ^b^Includes cigarettes, pipes, and cigars; ^c ^> 45 cigarettes/day; ^d^Gout flares and medications determined at baseline; ^e^Includes probenecid, allopurinol, and colchicine; ^f^Follow up from baseline to last observation. Mean (standard deviation) is provided unless specified otherwise. AMIS, Aspirin Myocardial infarction study; MI, myocardial infarction; NSAID, non-steroidal anti-inflammatory drug; sUA, serum urate; SD, standard deviation.

### Serum uric acid at baseline and cumulative incidence of outcomes

**Figure 1:**
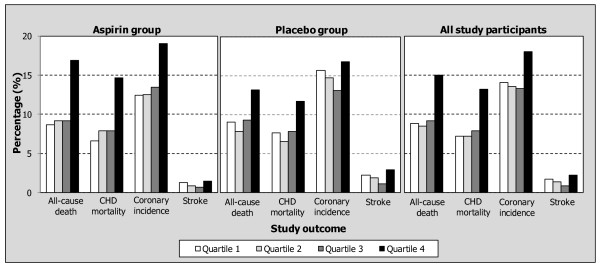
**Cumulative incidence of study endpoints according to baseline sUA concentration for patients receiving aspirin, placebo group, and all study participants: follow-up analysis of 4,352 participants in the AMIS Study**. Aspirin group: quartile 1-2.5 to 4.5 mg/dL for men, 1.4 to 5.25 mg/dL for women; quartile 2-4.53 to 5.37 mg/dL for men, 5.27 to 6.03 mg/dL for women; quartile 3-5.38 to 6.45 mg/dL for men, 6.05 to 7.0 mg/dL for women; quartile 4-6.47 to 10.45 mg/dL for men, 7.02 to 12.52 mg/dL for women. Placebo group: quartile 1-2.38 to 4.48 mg/dL for men, 1.83 to 5.28 mg/dL for women; quartile 2-4.5 to 5.42 mg/dL for men, 5.3 to 6.03 mg/dL for women; quartile 3-5.47 to 6.48 mg/dL for men, 6.05 to 6.97 mg/dL for women; quartile 4-6.52 to 11.05 mg/dL for men, 6.98 to 12.12 mg/dL for women. For all outcomes except stroke, the difference in incidence between the first and fourth quartiles and a linear trend test were statistically significant (*P *< 0.05). AMIS, Aspirin Myocardial Infarction Study; CHD, Coronary Heart Disease, sUA, serum urate.

Quartiles of sUA concentrations were defined following baseline measurements at randomization (Table 1). The mean sUA values for the aspirin and placebo groups were 6.15 ± 1.43 mg/dL (range 1.4 to 12.52 mg/dL) and 6.14 ± 1.35 mg/dL (range 1.83 to 12.02 mg/dL), respectively. Cumulative incidence of each outcome (all-cause death, CHD mortality, coronary incidence, and stroke) was highest in the fourth sUA quartile of each study group (aspirin, placebo, and all study participants; Figure 1). Notably, the cumulative incidence of all-cause death and CHD mortality was greater by 71% and 83%, respectively, from sUA quartile 1 to quartile 4. In each group, cumulative incidence of all-cause death, CHD mortality, and coronary incidence outcomes were statistically significantly higher in patients in the fourth quartile than in the respective first quartile (*P *< 0.05).

Cox regression models were used to calculate HRs per quartile of sUA for each outcome. Since there was no difference between the aspirin and placebo groups in terms of risk by sUA quartile, these data were subsequently pooled. Table 2 provides the estimated relative risk for each of the study outcomes. Patients in quartile 4 had the highest risk for each outcome, including an adjusted HR of 1.88 (95% CI, 1.45 to 2.46) for all-cause death, 1.99 (95% CI, 1.49 to 2.66) for CHD mortality, 1.36 (95% CI, 1.08 to 1.69) for coronary incidence, and 1.31 (95% CI, 0.69 to 2.49) for stroke. When sUA was analyzed as a continuous variable, each unit increase in sUA was associated with risk of all-cause death with an adjusted hazard ratio of 1.23 (1.15 to 1.31, *P *< 0.001), CHD mortality with a hazard ratio of 1.24 (1.16 to 1.33, *P *< 0.001), and coronary incidence with a hazard ratio of 1.12 (1.10 to 1.19, *P *< 0.01); this trend was not statistically significant for stroke (hazard ratio 1.08, 0.91 to 1.29, *P *= 0.37).

**Table 2:** Multivariable adjusted hazard ratio for each of the study outcomes stratified by quartile of serum uric acid concentration

	Hazard ratio (95% CI)			
	
	Quartile 1	Quartile 2	Quartile 3	Quartile 4
**Outcome**	***N *= 1,090**	***N *= 1,080**	***N *= 1,092**	***N *= 1,090**
All-cause death	1.00	0.96 (0.72, 1.29)	1.15 (0.87, 1.54)	1.88^a^ (1.45, 2.46)
CHD mortality	1.00	0.97 (0.71, 1.33)	1.17 (0.86, 1.60)	1.99^a^ (1.49, 2.66)
Coronary incidence	1.00	0.91 (0.72, 1.15)	0.96 (0.76, 1.21)	1.36^b^ (1.08, 1.69)
Stroke	1.00	0.65 (0.32, 1.34)	0.59 (0.27, 1.29)	1.31 (0.69, 2.49)

^a^*P *< 0.001 in comparison to the first quartile; ^b^*P *< 0.01. in comparison to first quartile; ^c^Trend test performed using serum urate as a continuous variable. The covariates adjusted for in this regression included: age, gender, African-American ethnicity, body mass index, blood pressure/hypertension, smoking, diabetes status, physical activity measure, hyperlipidemia, hypertriglyceridemia, renal function, and other non-steroidal anti-inflammatory drug use.

CHD, coronary heart disease; CI, confidence interval; N, number.

### Incidence of study outcomes by gout status

Records of all study participants were utilized for subgroup analysis of study outcomes by gouty arthritis. Mean baseline sUA was 6.06 ± 1.33 mg/dL in participants without gout, 7.18 ± 1.80 mg/dL in participants with gout and on gout medications, and 7.00 ± 1.66 mg/dL in participants with gout not receiving gout medication (*P *< 0.001 for all gout groups compared with patients without gout; Table 3).

**Table 3:** Multivariable adjusted hazard ratio for each study outcome by gout status

	Hazard Ratio (95% Confidence Interval)		
	All study participants		
**Gout status**	**No gout**	**Treated gout**	**Untreated gout**
	***N *= 3,987**	***N *= 129**	***N *= 89**
**Mean sUA (SD)**	**6.06 (1.33)**	**7.18 (1.80)**	**7.00 (1.66)**
All-cause death	1.00	0.84 (0.47, 1.50)	1.73^a ^(1.03, 2.91)
CHD mortality	1.00	0.82 (0.43, 1.54)	2.00^b ^(1.18, 3.36)
Coronary incidence	1.00	0.86 (0.58, 1.34)	1.67^a ^(1.07, 2.62)
Stroke	1.00	2.11 (0.74, 6.03)	0.83 (0.11, 6.04)

^a^*P *< 0.05 for comparison of untreated gout group to no gout group; ^b^*P *< 0.01 for comparison of untreated gout group to no-gout group. The covariates adjusted for in this regression included: age, gender, blood pressure/hypertension, smoking, diabetes status, physical activity measure, hyperlipidemia, hypertriglyceridemia, renal function, and other non-steroidal anti-inflammatory drug use. Gout medications accounted for in this study were allopurinol, probenecid, and colchicine. CHD, coronary heart disease; sUA, serum urate.

**Figure 2:**
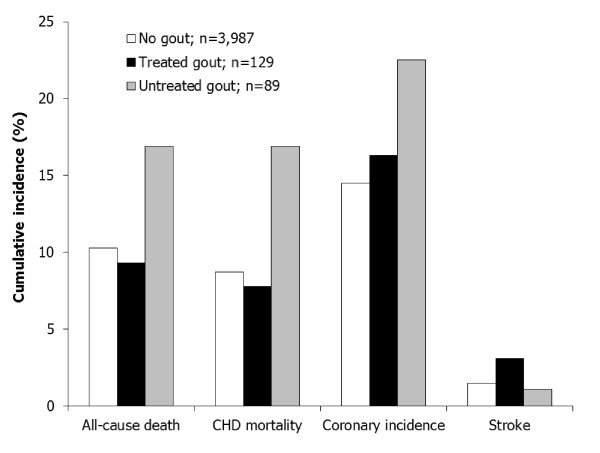
**Cumulative incidence of study endpoints by gout status**. For all outcomes except stroke, the rates among gout patients not on medication were significantly higher than for those without gout. Participants were considered to be treated for gout if they reported use of allopurinol, probenecid, or colchicine during the study. CHD: Coronary Heart Disease.

The cumulative incidence of study endpoints by gout status is presented in Figure 2. Participants with untreated gout experienced higher all-cause mortality (16.9% of study group), CHD mortality (16.9%) and coronary incidence (22.5%), compared to patients without gout or those who received gout medication.

Table 3 provides the results of secondary analysis on the relationship between gout medications and outcomes, as determined by Cox regression analysis. Serum urate was higher in the gout categories compared to the non-gout category (*P *< 0.01). Notably, risk for all study outcomes in patients treated with gout medications was indistinguishable from that of patients without gout. However, those with gout not receiving gout medications had significantly higher risk for all-cause death (HR 1.73 (95% CI, 1.03 to 2.91), *P *< 0.05), CHD mortality (HR 2.00 (95% CI, 1.18 to 3.36), *P *< 0.01), and coronary incidence (HR 1.67 (95% CI, 1.07 to 2.62), *P *< 0.05), compared with patients without evidence of gout.

## Discussion

The novelty of this study lies in the selection of the previously unstudied, yet high-risk, population of patients with existing coronary artery disease, and in the observed disparities in the outcomes with respect to gout treatments. Our observations that the patients with untreated gout have a significantly increased risk for all-cause death, CHD mortality, and coronary incidence and that participants with gout on treatment had risk comparable to those of participants without gout are important, yet must not be over-interpreted, given the modest sample size. As previously mentioned, the association between gout and the risk for CV outcomes is well recognized. The results reported here provide yet another reason for improving the treatment of gout [13,14].

Findings from this present study support the potential use of sUA as a biomarker for mortality outcomes in adult patients with pre-existing MI. Among participants of the AMIS Study, there is an association between sUA and risk of all-cause death, CHD mortality, and coronary incidence in individuals with a history of MI. Similar results were reported in a recent Israeli study, where, among 2,966 patients with documented CHD followed for 6.2 years, including those with a history of MI in the 6 months to 5 years prior to enrollment, there was a significant and positive correlation between the occurrence of fatal MI, nonfatal MI, or sudden cardiac death and increasing baseline sUA (*P *= 0.0009) [15]. These results also corroborate the results of the study by Ioachimescu *et al*. in which post-MI risk for poor outcomes was predicted by sUA [16].

Among patients with a history of heart failure (HF), a history of gout increased the risk of readmission due to HF or death by 63%, and recent acute gout (within 60 days) doubled the risk for HF readmission or death and increased the risk for all-cause mortality by 76%, after adjusting for confounders [17]. Of interest, we observed no association between sUA/gout and stroke outcomes, which is inconsistent with previous studies [18,19]. It is uncertain if this is due to the relatively small number of stroke events that occurred during the AMIS study [20].

At low doses (less than 1 g per day), aspirin reduces urate excretion in the kidney [21]. At the higher doses used in this study, aspirin is known to be uricosuric [22]. Since the two arms of the AMIS were systematically different all our initial analyses were done separately for each and the results were pooled only when the findings were comparable.

The causal relationship between uric acid, gout, and the pathogenesis of CHD is under investigation. A growing body of literature, comprehensively reviewed elsewhere [23], points toward the role of uric acid in promoting atherosclerosis through increased oxidative stress and inflammation in both gouty and asymptomatic hyperuricemic patients. *In vitro *studies have demonstrated that soluble uric acid promotes inflammation [24], generates intermediate reactive oxidative species [25] and leads to endothelial dysfunction and proliferation [26]. The presence of the urate transporter, URAT1, in human vascular smooth muscle cells [27] provides another link between uric acid and endothelial dysfunction, but studies are needed to determine if this and other polymorphisms responsible for hyperuricemia and gout are also linked to CHD. Several studies have suggested a pathogenic role for uric acid in hypertension and increased platelet adhesiveness and lysis [28]. Uric acid has been shown to promote oxygenation of low-density lipoprotein (LDL) cholesterol, and gout patients have significantly higher levels of oxidized LDL [23].

While the above evidence suggests a direct role for uric acid in the progression of CVD, other data suggest that the oxidative stress may be due to the activity of xanthine oxidase (XO), of which sUA is a marker. Urate lowering therapy (ULT) with allopurinol reduced the level of oxidized LDL in gout patients, while treatment with benzbromarone did not [29]. In patients with mild to moderate HF (mean sUA 7.12 mg/dL), allopurinol improved vascular blood flow (endothelium-dependent vasodilation) in a steep dose-dependent manner, while probenecid did not have this effect [30]. Three months of allopurinol treatment in hyperuricemic patients led to improvements in endothelial function directly related to the extent of sUA reduction, and this improvement was not seen in normouricemic controls treated with allopurinol [30]. In mice with experimental MI, treatment with allopurinol slowed down subsequent left ventricular remodeling (dilation, hypertrophy, and interstitial fibrosis), resulting in substantially improved left ventricular function. This effect was attributed to the inhibition of XO in the myocardium, leading to reduced oxidative stress [31]. Other studies have demonstrated improved CV outcomes when allopurinol was used in patients with documented CHD undergoing various interventions [32-34]. These data support the hypothesis that the oxidative species generated by XO activity contribute to the progression of CVD. However, recent work in newly hypertensive adolescents has demonstrated the beneficial effects of ULT with allopurinol [35]. It may be that both uric acid and XO are involved in the etiology of CVD through independent and common pathways. Regardless, since sUA is a direct reflection of XO activity, it serves as a useful biomarker for CVD risk.

Important caveats are due. First, the results on gout treatment and improved cardiovascular outcomes should be treated as hypothesis generating and not causative, since channeling bias (that is, getting treatment for gout might be a marker for healthier/health conscious individuals who may have better outcomes) can make it impossible to assess the true impact of ULT. Another piece of evidence suggesting a non-random 'allocation' of therapy might be the serum urate concentrations. The fact that serum urate concentration is not lower in the treated group suggests that these individuals had higher serum urate/more severe gout to begin with. With such a bias, the mortality benefit associated with urate lowering might be an underestimate. Another data-related issue is our inability to exclude colchicine from urate lowering therapies. Our study examined risk factors for coronary events among those who survived such an event in the past. The influence of survivor effect and the importance of lifestyle modification following a diagnosis of coronary artery disease is difficulty to model in our study. Another important facet of this study is that it was performed in the 1970s, before the advent of current sophisticated diagnostic tools to detect subtle coronary syndromes. The patients with MI included in the trial are likely to be survivors from a more definitive, more severe, acute myocardial infarction. In that context there is a survival bias in that these individuals are likely to be hardier than those who survive a non-ST-elevation acute myocardial infarction. Nevertheless, the outcome we studied, mortality, was applied uniformly across all strata of urate levels as well as gout status, and arguably there were no differential biases.

Other factors can be expected to attenuate the relationship between hyperuricemia and gout and coronary events. When multiple risk factors contribute to the risk of an outcome, the risk factors for the recurrence of the event tend to be correlated with each other leading to index event bias. This bias could have resulted in the underestimation of the true relationship between hyperuricemia and gout and coronary events in our study. Our study data did not have the granularity or statistical power to discern any differences by the type of gout medication used. Finally, the effect of residual confounding by unobserved and unadjusted risk factors could not be estimated in our study.

## Conclusions

The data presented here suggest that hyperuricemia is an independent predictor of all-cause and CHD mortality, as well as coronary incidence, in individuals with confirmed CHD. Analysis of sUA levels in patients following MI may be a reliable and inexpensive method for distinguishing patients at risk for mortality. Measurement of sUA levels may contribute to existing strategies that stratify risk for secondary CHD events, including mortality, in patients with previous MI.

## Abbreviations

ACS: acute coronary syndrome; AMIS: Aspirin Myocardial Infarction Study; CHD: coronary heart disease; CI: confidence interval; CRP: C-reactive protein; CVD: cardiovascular disease; HR: hazard ratio; LDL: low density lipoprotein; MI: myocardial infarction; NHLBI: National Heart Lung Blood Institute; sUA: serum urate; ULT: urate lowering therapy; XO: xanthine oxidase.

## Competing interests

Eswar Krishnan has received honoraria, research grants, ad-board fees or consulting fees from the following entities: Ardea Biosciences, URL Pharma, Metabolex, Takeda Pharmaceuticals and Savient Pharmaceuticals. Dr Krishnan has held common stocks for Savient Pharmaceuticals in the recent past. Drs Hariri and Dabbous are employees of Takeda, Inc. Dr Pandya was an employee of Takeda at the time of performance of this work.

Dr Lingala has no conflicts of interest to declare.

Proprietary products manufactured by these companies are not discussed in this manuscript.

## Authors' contributions

The data used for these analyses were obtained from the NHLBI Limited Access program. EK conceived the manuscript idea, designed the analysis plan, performed statistical analysis, interpreted the results, and wrote the first draft of the manuscript with assistance from other authors. He has possession of raw data sets and takes responsibility for the integrity of the data and the accuracy of the data analysis. Takeda Pharmaceuticals International, Inc. did not have access to the raw data and Takeda authors contributed primarily to refinement of study design, interpretation of data, editing, and revisions to the initial drafts. Editing and bibliography assistance on earlier versions of this manuscript was provided by Kristen Wieghaus Quinn, PhD, and Meryl Gersh, PhD, of AlphaBioCom, LLC, in Radnor, PA, and was funded by Takeda Pharmaceuticals, Inc. The authors had complete authority over the content and received no financial remuneration for the article. All authors have read and approved the manuscript for publication.
